# A first trimester prediction model and nomogram for gestational diabetes mellitus based on maternal clinical risk factors in a resource-poor setting

**DOI:** 10.1186/s12884-024-06519-7

**Published:** 2024-05-06

**Authors:** Bruno Basil, Izuchukwu Nnachi Mba, Blessing Kenechi Myke-Mbata, Simeon Adelani Adebisi, Efosa Kenneth Oghagbon

**Affiliations:** 1 Department of Chemical Pathology, Benue State University, Makurdi, Nigeria; 2 Department of Chemical Pathology, Nile University of Nigeria, Abuja, Nigeria

**Keywords:** Body mass index, First trimester, Foetal macrosomia, Gestational diabetes mellitus, Maternal clinical risk factors, Nomogram, Prediction model

## Abstract

**Background:**

The implementation of universal screening for Gestational Diabetes Mellitus (GDM) is challenged by several factors key amongst which is limited resources, hence the continued reliance on risk factor-based screening. Effective identification of high-risk women early in pregnancy may enable preventive intervention. This study aimed at developing a GDM prediction model based on maternal clinical risk factors that are easily assessable in the first trimester of pregnancy in a population of Nigerian women.

**Methods:**

This was a multi-hospital prospective observational cohort study of 253 consecutively selected pregnant women from which maternal clinical data was collected at 8–12 weeks gestational age. Diagnosis of GDM was made via a one-step 75-gram Oral Glucose Tolerance Test (OGTT) at 24–28 weeks of gestation. A GDM prediction model and nomogram based on selected maternal clinical risk factors was developed using multiple logistic regression analysis, and its performance was assessed by Receiver Operator Curve (ROC) analysis. Data analysis was carried out using Statistical Package for Social Sciences (SPSS) version 25 and Python programming language (version 3.0).

**Results:**

Increasing maternal age, higher body mass index (BMI), a family history of diabetes mellitus in first-degree relative and previous history of foetal macrosomia were the major predictors of GDM. The model equation was: LogitP = 6.358 − 0.066 × Age − 0.075 × First trimester BMI − 1.879 × First-degree relative with diabetes mellitus − 0.522 × History of foetal macrosomia. It had an area under the receiver operator characteristic (ROC) curve (AUC) of 0.814 (95% CI: 0.751–0.877; p-value < 0.001), and at a predicted probability threshold of 0.745, it had a sensitivity of 79.2% and specificity of 74.5%.

**Conclusion:**

This first trimester prediction model reliably identifies women at high risk for GDM development in the first trimester, and the nomogram enhances its practical applicability, contributing to improved clinical outcomes in the study population.

**Supplementary Information:**

The online version contains supplementary material available at 10.1186/s12884-024-06519-7.

## Introduction

Gestational Diabetes Mellitus (GDM) poses a significant health concern during pregnancy, impacting both maternal and foetal outcomes [1]. While its prevalence is increasing globally and in Africa [2, 3], resource-poor countries face unique challenges in managing and preventing this condition. In Nigeria, a highly populated country in sub-Saharan Africa with a higher proportion of its people in the sub-urban and rural areas, the burden of GDM is high [4], necessitating targeted and practical approaches for early identification and intervention.

Gestational Diabetes Mellitus (GDM), one of the most common complications of pregnancy, arises due to alterations in maternal glucose metabolism and insulin sensitivity which occurs as a result of physiological changes during pregnancy for which the beta-cells of the pancreas are unable to compensate [5, 6]. In the early stages of pregnancy, this hyperglycemia stimulates increased expression of parathyroid hormone-related protein (PTH-rP) and its receptor (PTH-R1), along with vascular endothelial growth factor (VEGF) and CD31 leading to substantial disruptions in placental function and angiogenesis, which are crucial for maintaining a healthy feto-maternal environment during pregnancy, potentially leading to adverse pregnancy outcomes [7, 8]. Considering the impact of GDM and its complications on both maternal and fetal well-being, the need for universal screening via Oral Glucose Tolerance Test (OGTT) becomes evident. Due to its proven benefit over risk factor-based screening, universal screening is highly recommended, even in early pregnancy, given availability of financial, material, space, and human resources [9, 10]. However, implementing oral glucose tolerance testing during pregnancy for all women, and more than once in some, is both operationally challenging and costly, hence the continued emphasis on the need to improve risk factor-based screening approach in most developing countries especially in sub-Saharan Africa.

The first trimester of pregnancy has been explored as a crucial window for predicting the risk of GDM development [11, 12]. This is necessary because hyperglycaemia in early pregnancy may induce pathologic changes in the foetus prior to the diagnosis of GDM later in pregnancy [13, 14]. However, existing first trimester prediction models, which often involve elaborate biochemical testing [15–17], stem predominantly from high-income settings and may not be directly applicable or feasible in resource-constrained environments. Also, certain parameters needed for effective application of the current suggested models may be unavailable, come at prohibitive costs or result in a delay in decision making. Therefore, there is a pressing need for a setting-specific prediction model that relies on easily accessible maternal clinical risk factors, allowing for easy implementation even in primary care settings with limited healthcare resources.

This study aimed at developing a GDM prediction model based on maternal clinical risk factors easily assessable in the first trimester of pregnancy in a cohort of Nigerian women with singleton pregnancies. It involved the development of a maternal clinical risk factors-based prediction model and a user-friendly nomogram that is easily interpretable and implementable, to facilitate timely interventions and improved outcomes for both mothers and their infants. The study also aims at contributing to mitigating the impact of GDM in sub-Saharan Africa and similar settings worldwide where there is a paucity of studies on early prediction of the disease.

## Methods

### Study design and setting

This was a multihospital-based cohort study of 253 consecutively selected women at 8–12 weeks gestational age. These women received antenatal care at Federal Medical Centre (FMC), Benue State University Teaching Hospital (BSUTH), First Fertility Hospital, Family Support Program and Pishon Women Hospital; all these centres were located in Makurdi, the capital city of Benue state, Nigeria. Recruitment of participant and first trimester data collection were performed between June 2018 and June 2019 while the follow-up of the participants and subsequent OGTT was performed between September 2018 and March 2020.

### The cohort

**Fig. 1:**
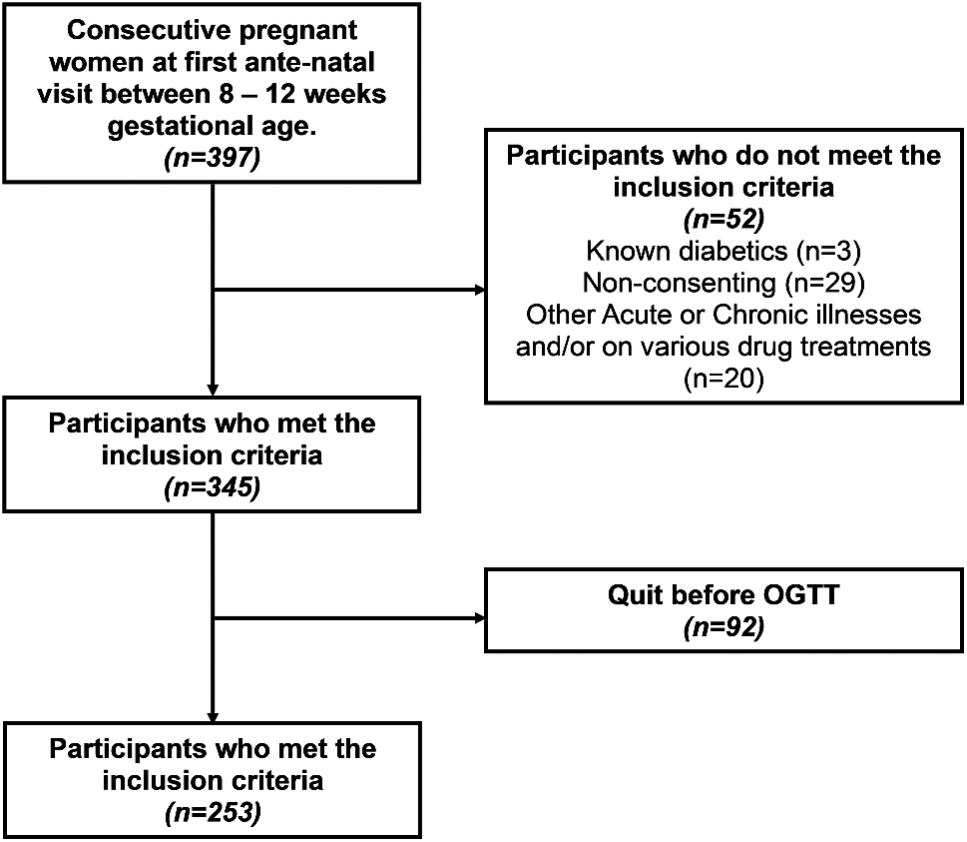
Flow chart showing recruitment of participants

Pregnant women aged 18–45 years, in their first trimester, with ultrasonographically confirmed singleton pregnancies, and from whom informed and written consent were obtained, were considered to have met the inclusion criteria for the study. Those with pre-existing diabetes mellitus (DM), multiple gestation, known acute or chronic illness (e.g., active infection or inflammatory conditions, severe hypertension, ischemic heart disease, chronic kidney disease, polycystic ovary syndrome, Cushing’s disease, thyroid disorders, etc.), or on any medications affecting glucose metabolism (e.g., corticosteroids) were excluded. Participants were followed through pregnancy, and only those who were later tested for GDM by using a one-step 75 g OGTT at 24–28 weeks of gestation were included in the study (Fig. 1). Findings from group of participants who developed GDM were compared against those who had normal pregnancy (non-GDM) during statistical analysis. The minimum sample size was calculated based on the previously reported prevalence of GDM in Nigerian [4], a significance level of 0.05, and an adjustment for a 10% non-response rate. The target minimum sample size was calculated to be 173 participants. However, a total of 253 participants who were recruited completed the study.

### Data collection

Clinical data were collected from eligible participants during their first antenatal visit (8 to 12 weeks). Demographic information, medical history, and anthropometric measurements were obtained. Several maternal clinical risk factors were considered for the prediction model, including maternal age, body mass index (BMI), family history of diabetes, parity, blood pressure, etc. These factors were chosen based on their established associations with GDM in the literature [18, 19]. 

### Testing for GDM

The follow-up after the recruitment of each participant lasted for an average of 12 weeks. At 24–28 weeks of gestation, participants underwent an OGTT with an oral load of 75 g of anhydrous glucose. This was administered after ensuring that participants maintained a normal diet and activity for at least 3 days prior to testing. Samples for plasma glucose estimation were collected into tubes containing fluoride oxalate, centrifuged within 20 min of collection and analyzed in batches immediately after collection. GDM was diagnosed based on International Association of Diabetes and Pregnancy Study Groups (IADPSG) criteria [20]. A diagnosis of GDM was established if the fasting blood glucose level was ≥ 5.1 mmol/L, 1-h post-75 g blood glucose level was ≥ 10.0 mmol/L, or 2-h post-75 g blood glucose level was ≥ 8.5 mmol/L.

### Statistical analysis

Data analysis was performed using the Statistical Package for Social Sciences (SPSS) version 25 from IBM corporation, Armonk, New York, USA. Descriptive statistics were used to summarize the demographic and clinical characteristics of the study population. Univariate analyses were performed to identify significant associations between maternal clinical risk factors and the development of GDM. Subsequently, a multivariable logistic regression model was used to assess the independent contribution of each risk factor.

To ensure the reliability and generalizability of the logistic regression model, the model was initially trained and adjusted on a dedicated training set, and its predictive performance was subsequently evaluated on an independent learning set. This process of internal validation allowed for the assessment of the stability and variability of the model coefficients. A nomogram was developed based on the model coefficients obtained from the logistic regression analysis using the matplotlib library in Python programming language (version 3.0) from Python Software Foundation (PSF). This provided a visual representation of the model to allow for easy estimation of an individual’s risk of developing GDM. The model’s performance was evaluated using measures such as the predictive probability, sensitivity, specificity, and area under the receiver operating characteristic (ROC) curve. The Hosmer-Lemeshow goodness-of-fit test for the observed data range was used to calibrate this model, and values greater than 0.05 indicated a good fit. Statistical significance level was set at a p-value of < 0.05.

## Results

A total of 345 pregnant women were recruited to this study but only 253 underwent a 75-g OGTT for GDM evaluation and were included in the analysis. The results from the procedure led to the diagnosis of GDM in 52 (20.6%) participants, while 201 (79.4%) were normoglycemic pregnancies. Table 1 shows the maternal demographic and clinical characteristics of the cohort with selected risk factors by GDM and non-GDM participants. Compared with the non-GDM women, those who developed GDM were significantly older (31.4 ± 4.6 years versus 28.8 ± 4.7 years; p-value < 0.001), had higher BMI (31.8 ± 5.5 kg/m2 versus 27.7 ± 4.7 kg/m2; p-value < 0.001) and were significantly more likely to have first-degree relatives with diabetes mellitus (46% versus 10%; p-value < 0.001) and a history of foetal macrosomia in previous pregnancies (67.3% versus 6.5%; p-value < 0.001).

**Table 1:** Maternal demographics and clinical characteristics by GDM and Non-GDM pregnant women in Makurdi, North-West, Nigeria (*n* = 253)

Variables	Non-GDM women (*n* = 201) Mean ± SD or n (%)	GDM women (*n* = 52) Mean ± SD or n (%)	p-values
Maternal age (years)	28.8 ± 4.7	31.4 ± 4.6	< 0.001*
Gestational age at enrolment (weeks)	10.1 ± 1.2	9.7 ± 1.1	0.059
BMI at enrolment (kg/m^2^)	27.7 ± 4.7	31.8 ± 5.5	< 0.001*
Educational status * Primary* * Secondary* * Tertiary* * Uneducated*	41 (20.4) 40 (19.9) 66 (32.8) 54 (26.9)	9 (17.3) 10 (19.2) 23 (44.2) 10 (19.2)	0.443
Occupational status * Unemployed* * Civil servant* * Trading* * Farming* * Student* * Others*	29 (14.4) 64 (31.8) 25 (12.4) 47 (23.4) 25 (12.4) 11 (5.5)	6 (11.5) 20 (38.5) 5 (9.6) 16 (30.8) 4 (7.7) 1 (1.9)	0.566
Marital status * Married* * Single*	173 (86.1) 28 (13.9)	42 (80.8) 10 (19.2)	0.384
Ethnicity * Tiv/Etulo* * Idoma/Igede* * Igbo* * Others*	108 (53.7) 65 (32.3) 26 (12.9) 17 (8.5)	31 (59.6) 15 (28.8) 4 (7.7) 2 (3.8)	0.450
Religion * Christianity* * Islam* * Others*	170 (84.6) 26 (12.9) 5 (2.5)	44 (84.6) 6 (11.5) 2 (3.8)	0.844
Parity * Primigravida* * Multigravida* Previously screened for GDM Not previously screened for GDM	70 (34.8) 131 (65.2) 16 (8.0) 115 (57.2)	12 (23.1) 40 (76.9) 3 (5.8) 37 (71.1)	0.135
Blood Pressure * Systolic (SBP)* * Diastolic (DBP)*	119.7 ± 8.9 78.9 ± 5.6	121.3 ± 7.9 80.2 ± 4.7	0.252 0.117
Risk Factors for GDM			
History of Gestational hypertension	32 (15.9)	11 (21.2)	0.082
First-degree Relative with DM	20 (10.0)	24 (46.2)	< 0.001*
History of Pre-term deliveries	44 (21.9)	13 (25.0)	0.530
History of GDM	0 (0.0)	3(5.8)	< 0.001*
History of Peri-natal losses	21 (10.5)	9 (17.3)	0.349
History of Multiple pregnancies	6 (11.5)	3 (1.5)	0.065
History of Foetal macrosomia	19 (6.5)	35 (67.3)	0.019*
History of Pre-eclampsia	10 (5.0)	0 (0.0)	0.119

*p-value significant at 0.05; SD – Standard deviation; n – Number of observations within the category; GDM – Gestational diabetes mellitus; BMI – Body mass index

**Fig. 2:**
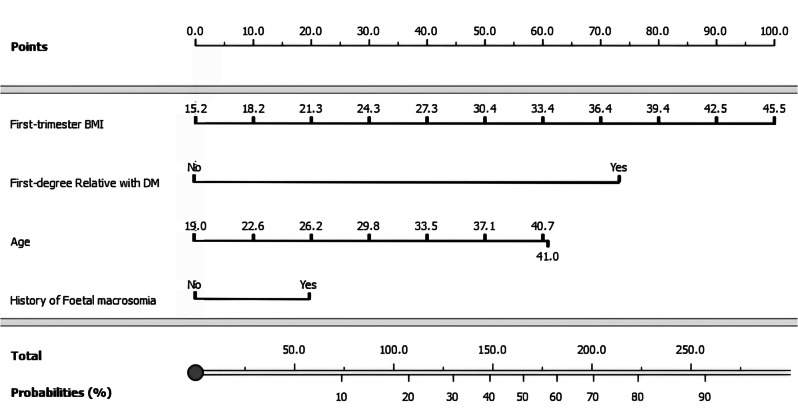
Nomogram to estimate the risk of GDM development. Each predictor is assigned a score on each axis. Compute the sum of points for all predictors and denote this value as the total points. The corresponding “risk of GDM” of “total point” was converted to a predicted probability of GDM in percentage

A multivariable logistic regression analysis was used to establish a predictive model for GDM risk in the study population, as outlined in Table 2. The model equation was: *LogitP = 6.358 − 0.066 × Age − 0.075 × First trimester BMI − 1.879 × First-degree relative with DM − 0.522 × History of foetal macrosomia*. The initial model (AUC = 0.827; 95% CI: 0.712–0.942; p-value < 0.001) was employed for training and adjustments (internal validation). Subsequently, the model derived from the trained set (AUC = 0.814; 95% CI: 0.751–0.877; p-value < 0.001) demonstrated consistent performance when applied to the learning set (AUC = 0.816; 95% CI: 0.743–0.890, p-value < 0.001) supporting the potential utility of the validated model in predicting gestational diabetes mellitus (GDM) risk. A user-friendly and implementable scoring system for identifying high-risk women in clinical practice was established through the derivation of a nomogram from the predictive model (Fig. 2).

**Table 2:** Multivariable logistic regression for establishment of GDM predictive model based on maternal demographic and clinical risk factors during first trimester of pregnancy (*n* = 253)

Variables in the model	Category	Coefficients	OR (95% CI)	p-value
Age	Continuous	− 0.066	0.936 (0.864–1.014)	0.010
First trimester BMI	Continuous	− 0.075	0.928 (0.857–1.005)	0.005
First-degree Relative with DM	Binary: Yes = 1; No = 2	− 1.879	0.153 (0.073–0.318)	< 0.001
History of Foetal macrosomia	Binary: Yes = 1; No = 2	− 0.522	0.593 (0.266–1.324)	0.047
Constant		6.358		

*p-value significant at 0.05; OR – Odds Ratio; CI – Confidence Interval; DM – Diabetes mellitus; BMI – Body mass index

A notable finding was the development of GDM in all women who had a previous history of GDM, 5.8% (*n* = 3; p-value < 0.001). However, this was not included in the prediction matrix due to its inaccessibility in the study population as most women were not screened for GDM in their previous pregnancies.

**Fig. 3:**
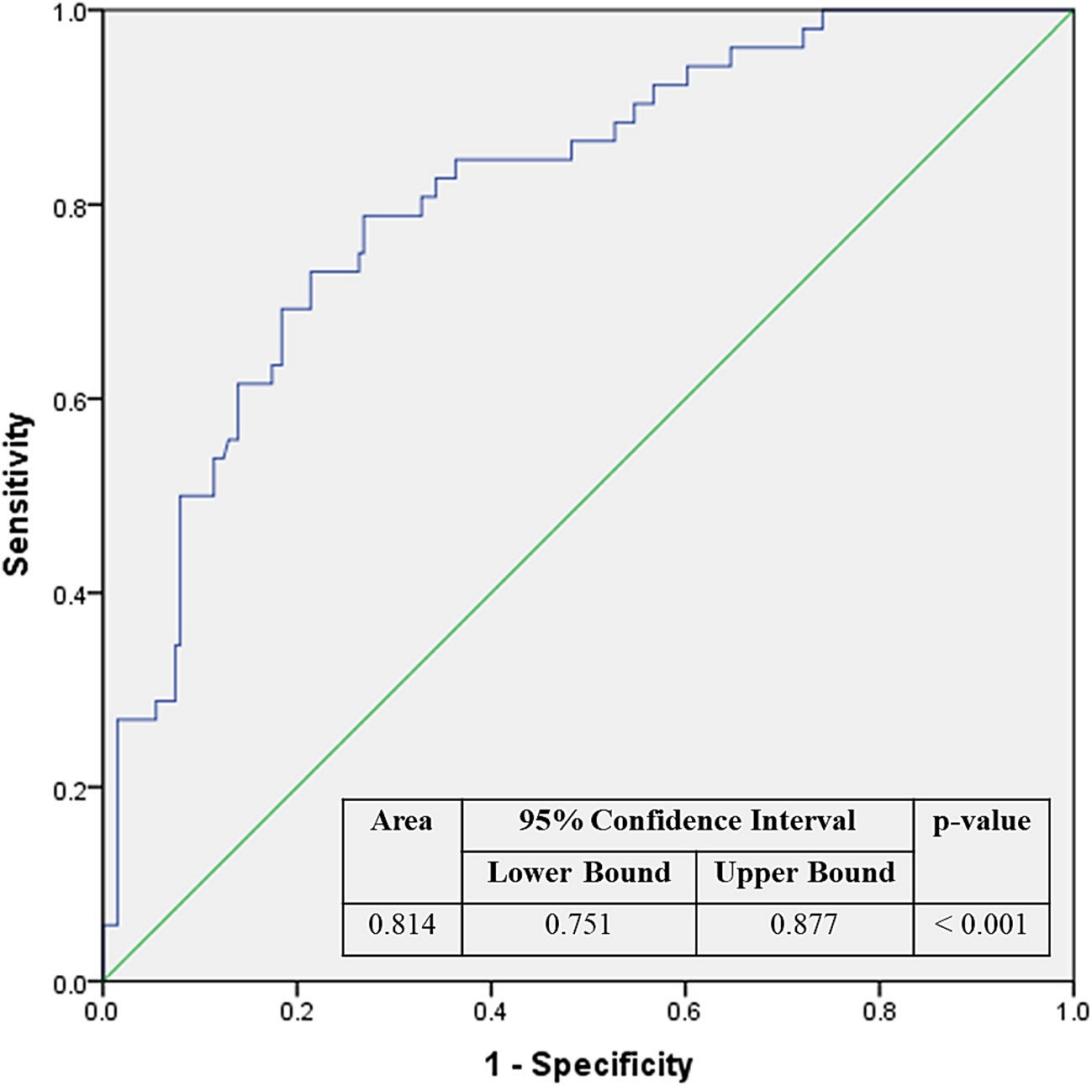
Receiver operator curve for maternal clinical risk factors-based prediction model for GDM development

The diagnostic performance of the prediction model in the cohort was assessed by ROC analysis (Fig. 3). It had a strong diagnostic accuracy with an area under the ROC curve (AUC) of 0.814 (95% CI: 0.751–0.877; p-value < 0.001). At the predicted probability threshold of 0.745 associated with the maximized Youden’s index of 0.537 and a selected cut-off of 0.5, the model had a sensitivity of 79.2% and specificity of 74.5%, demonstrating a well-accepted predictive and discriminative performance. Hosmer-Lemeshow goodness-of-fit test of 0.595 indicates that the model’s predicted probabilities correspond with the actual outcomes.

## Discussion

In this study, increasing maternal age, higher BMI, a family history of diabetes mellitus in first-degree relative and previous history of foetal macrosomia were found to be the major predictors of GDM in the study population. These factors coupled with the increased risk due to ethnicity (black race) were mostly responsible for the high incidence of GDM in this homogenous cohort. Although there were no significant differences in GDM risk amongst the various tribes in the study population, their uniform racial and ethnic features predisposed them to high risk of GDM.(21).

Previous studies have established the pathological basis for the existence of a strong link between increasing maternal age and higher BMI. This may be due to an age-related mitochondrial dysfunction leading to increased production of reactive oxygen species (ROS) which impair the insulin receptor complex in skeletal muscles amongst many other mechanisms [22], as well as the obesity-induced adipose tissue dysfunction and subclinical inflammation [23], which lead to insulin resistance. Also, having a family history of diabetes mellitus and a previous history of foetal macrosomia have also been found to be significant independent predictors of GDM in previous studies carried out in similar populations within the region [3, 24]. The family history of diabetes mellitus may suggest genetic predisposition to GDM while the history of foetal macrosomia may indicate a possible presence of GDM in the previous pregnancy.

The significant risk factors for GDM from our univariate analysis were included in a predictive model. However, a notable finding in this study is the exclusion of the history of gestational diabetes mellitus (GDM) from the predictive model, despite its significance in the univariate analysis. This is due to the absence of universal screening for GDM in our study area which renders the extraction of reliable historical data challenging. The aim of the study which is to assess the predictive performance of easily accessible maternal clinical risk factors in the first trimester for GDM makes practical considerations like this important. The small sample size of participants with a GDM history (*n* = 3) further complicated the inclusion, raising concerns about the potential bias introduced by this limited representation.

The model in this study compares with previously suggested models in a recent systematic review with acceptable discriminatory ability (AUCs > 0.7) highlighting the reliability and efficacy of our model in predicting GDM risk during the first trimester [11]. Similar to our findings, maternal age, BMI, family history of DM, and history of GDM were the common major predictors in most studies. However, our study excluded the history of GDM while including the history of foetal macrosomia, which often associated with GDM [25], may function as a surrogate marker. Unlike most previous studies employing formula-given models, this study employed a user-friendly visual representation (nomogram) which enhances its practical utility in clinical settings, simplifying risk assessment for healthcare providers. Also, a recent Tanzanian study demonstrated better performance (AUC = 0.970) than our model [26]. This exceptional performance may be due to the use of mid-upper arm circumference (MUAC) and body fat percentage which are better indicators of fat mass than BMI as used in our model. They also ensured enhanced clinical utility of the model by developing a risk score. However, despite its promising nature, the unavailability of a bioelectrical impedance analyzer in most ANC settings in the region limits its application.

Previous studies assessed various early pregnancy prediction models including use of foetal heart rate, maternal clinical parameters and biochemical markers of which some had higher AUC compared to the index study [27–29], but these were mostly in non-African populations and may not be applicable in resource-constrained environments. In the sub-Saharan African region, there is a scarcity of studies on first-trimester prediction of GDM. A prior Nigerian study, employing the biochemical predictor sex hormone-binding globulin (SHBG), demonstrated superior predictive ability (AUC = 0.874) compared to our model (AUC = 0.814) [12]. However, the practical applicability of the SHBG model is compromised by limited accessibility of SHBG assay in many prenatal centers especially in rural and suburban areas, lack of standardization of the assay, its high cost and prolonged turn-around times. Despite being slightly less predictive, our model prioritizes practical feasibility, considering our study objectives.

Our study has some limitations that warrant consideration for future research and application. Firstly, variations in demographic characteristics and risk factor prevalence among study populations may influence model performance. As our study is specific to a Nigerian population, some risk factors might have varying degrees of influence in different populations emphasizing the need for cautious interpretation in diverse settings. Additionally, the inclusion of clinical variables in models varies, and our focus on easily accessible maternal clinical risk factors might differ from variables included in other models, affecting overall comparability. Also, differences in procedures for GDM diagnosis, including variations in glucose tolerance tests, can contribute to performance variations, emphasizing the importance of standardization, and expansion of the study to a wider, multi-ethnic scale could enhance the model’s generalizability. A shared limitation with many other existing models is the need for extensive external validation, emphasizing the necessity of validating the model in diverse populations to ensure its broader applicability, particularly in resource-poor settings.

## Conclusion

In this study, we developed a first trimester prediction model based on key maternal clinical risk factors that are easily accessible in a population of Nigerian pregnant women. The model showed a satisfactory diagnostic performance, and the accompanying nomogram enhances its practical utility, promising improved clinical outcomes by identifying high-risk women, particularly in resource-poor settings were facilities for implementation of universal screening are limited.

### Electronic supplementary material

Below is the link to the electronic supplementary material.

Supplementary Material 1

## Data Availability

The datasets used during the current study will be available on request to the corresponding author. This is because the data set contains other data that are unrelated to this study and may need to be excluded.

## Abbreviations

GDM: Gestational Diabetes Mellitus
OGTT: Oral Glucose Tolerance Test
PTH-rP: Parathyroid hormone-related protein
PTH-R1: PTH-rP receptor
VEGF: Vascular endothelial growth factor
ROC: Receiver Operating Characteristic
AUC: Area Under the Curve
SPSS: Statistical Package for the Social Sciences
DM: Diabetes Mellitus
FMC: Federal Medical Centre
BSUTH: Benue State University Teaching Hospital
BMI: Body Mass Index
IADPSG: International Association of the Diabetes and Pregnancy Study Groups
SD: Standard Deviation
OR: Odds Ratio
CI: Confidence Interval
MUAC: Mid-Upper Arm Circumference
SHBG: Sex Hormone-Binding Globulin
