# Supplementary figures and images for: A first trimester prediction model and nomogram for gestational diabetes mellitus based on maternal clinical risk factors in a resource-poor setting
Source: BMC Pregnancy Childbirth. 2024 May 6;24:346. doi: 10.1186/s12884-024-06519-7 (PMC11071227; doi:10.1186/s12884-024-06519-7)

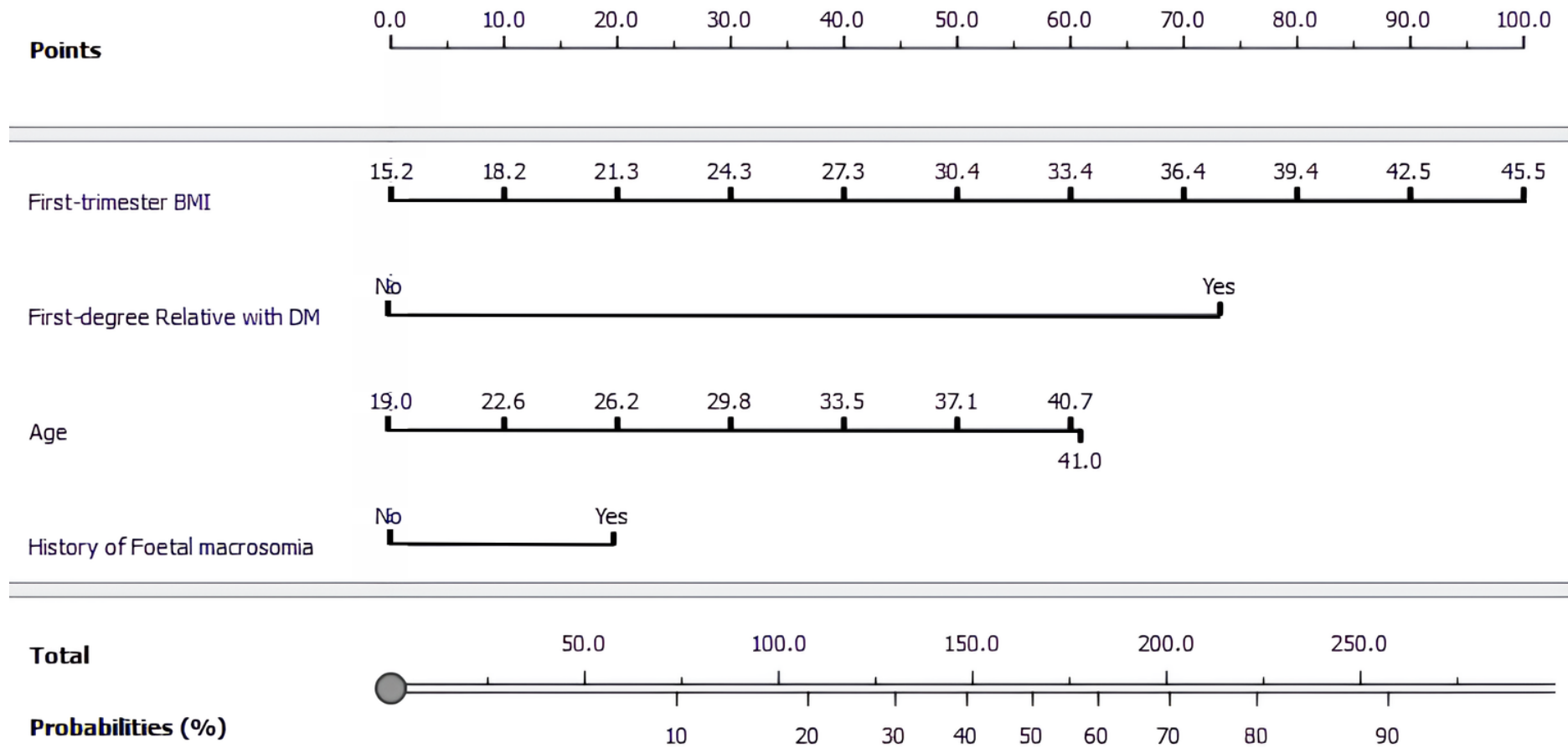

Supplement: Supplementary file 1 — Supplementary Material 1 [file 12884_2024_6519_MOESM1_ESM.pdf]
